# A randomised placebo-controlled trial of the effectiveness of early metformin in addition to usual care in the reduction of gestational diabetes mellitus effects (EMERGE): study protocol

**DOI:** 10.1186/s13063-022-06694-y

**Published:** 2022-09-21

**Authors:** F. Dunne, C. Newman, D. Devane, A. Smyth, A. Alvarez-Iglesias, P. Gillespie, M. Browne, M. O’Donnell

**Affiliations:** 1 Department of Medicine, HRB Clinical Research Facility, National University of Ireland Galway, Galway, Ireland; 2 HRB-Trials Methodology Research Network, National University of Ireland Galway, Galway, Ireland; 3 School of Nursing and Midwifery, National University of Ireland, Galway, Ireland; 4 Evidence Synthesis Ireland, National University of Ireland Galway, Galway, Ireland; 5 Cochrane Ireland, National University of Ireland Galway, Galway, Ireland; 6 Health Economics & Policy Analysis Centre (HEPAC), Institute for Lifecourse and Society (ILAS), National University of Ireland Galway, Galway, Ireland; 7 CÚRAM, the SFI Research Centre for Medical Devices (12/RC/2073_2), National University of Ireland Galway, Galway, Ireland

**Keywords:** Metformin, Gestational diabetes mellitus, Pregnancy, Randomised controlled trial

## Abstract

**Background:**

Pregnancies affected by gestational diabetes mellitus (GDM) are associated with an increased risk of adverse maternal and foetal outcomes. Current treatments for GDM involve initial medical nutritional therapy (MNT) and exercise and pharmacotherapy in those with persistent hyperglycaemia. Insulin is considered first-line pharmacotherapy but is associated with hypoglycaemia, excessive gestational weight gain (GWG) and an increased caesarean delivery rate. Metformin is safe in selected groups of women with GDM but is not first-line therapy in many guidelines due to a lack of long-term data on efficacy. The EMERGE trial will evaluate the effectiveness of early initiation of metformin in GDM.

**Methods:**

EMERGE is a phase III, superiority, parallel, 1:1 randomised, double-blind, placebo-controlled trial comparing the effectiveness of metformin versus placebo initiated by 28 weeks (+6 days) plus usual care.

Women aged 18–50 years will be recruited. Women with established diabetes, multiple pregnancies, known major congenital malformation or small for gestational age (<10th centile), intolerance or contraindication to the use of metformin, shock or sepsis, current gestational hypertension or pre-eclampsia, significant gastrointestinal problems, congestive heart failure, severe mental illness or galactose intolerance are excluded.

**Intervention:**

Immediate introduction of metformin or placebo in addition to MNT and usual care. Metformin is initiated at 500mg/day and titrated to a maximum dose of 2500mg over 10 days. Women are followed up at 4 and 12 weeks post-partum to assess maternal and neonatal outcomes.

The composite primary outcome measure is initiation of insulin or fasting blood glucose ≥ 5.1 mmol/L at gestational weeks 32 or 38. The secondary outcomes are the time to insulin initiation and insulin dose required; maternal morbidity at delivery; mode and time of delivery; postpartum glucose status; insulin resistance; postpartum body mass index (BMI); gestational weight gain; infant birth weight; neonatal height and head circumference at delivery; neonatal morbidities (neonatal care unit admission, respiratory distress, jaundice, congenital anomalies, Apgar score); neonatal hypoglycaemia; cost-effectiveness; treatment acceptability and quality of life determined by the EQ5D-5L scale.

**Discussion:**

The EMERGE trial will determine the effectiveness and safety of early and routine use of metformin in GDM.

**Trial registration:**

EudraCT Number 2016-001644-19l; NCT NCT02980276. Registered on 6 June 2017.

**Supplementary Information:**

The online version contains supplementary material available at 10.1186/s13063-022-06694-y.

## Administrative information

**Table:** 

Title	**A Randomised Placebo-Controlled Trial of the effectiveness of Early Metformin in Addition to Usual Care in the Reduction of Gestational Diabetes Mellitus Effects (EMERGE): Study Protocol**
**Trial registration**	EudraCT Number: 2016-001644-19l; NCT NCT02980276, registered 6/6/2017; https://clinicaltrials.gov/ct2/show/NCT02980276
**Protocol version**	EMERGE protocol version 8 (27^th^ July 2021) is currently in use
**Author details**	PG: Health economist whose research activity is focused on applying the techniques of economic evaluation to inform health policy and practice. PG is responsible for the study development, protocol preparation and protocol revision. FD: Clinical Endocrinologist and clinical trial investigator involved as Chief Investigator of EMERGE. FD conceived the trial, secured funding through competitive peer review process, enrolled participants to EMERGE, constructed and drafted this protocol manuscript. AAI: Statistical considerations related to sample size calculations, writing of the statistical analysis plan, processing and analysis of data for Data Monitoring Committees and creation of final technical report. AS: Experienced clinical trial methodologist involved in drafting the trial protocol, case report forms, design of clinical trial database and approval of study-specific documents from the Sponsor perspective. Medical monitor throughout EMERGE trial addressing clinical trial queries from trial sites and review of critical data points and safety metrics. DD: Midwife and trial methodologist involved in drafting the trial protocol and member of core team. CN: Clinical Endocrinologist and co- PI of EMERGE, reviewed and edited this manuscript. MB: CRF Clinical Trials Programme Manager, contributed to drafting of the trial protocol, reviewed and edited this manuscript. MOD: Experienced clinical trial methodologist involved in the design of the trial, involved in drafting the trial protocol and approval of study-specific documents and a member of the core team. Reviewed and edited this manuscript.
**Name and contact information for the trial sponsor**	*National University of Ireland, Galway*
**Role of sponsor**	The study sponsor and funder had no role in the study design; collection, management, analysis, and interpretation of data; writing of the report; or the decision to submit the report for publication

## Introduction

### Background and rationale

Gestational diabetes mellitus (GDM) is defined as carbohydrate intolerance resulting in hyperglycaemia of variable severity with onset or first recognition during pregnancy, excluding those with overt diabetes [1]. The International Association for the Study of Diabetes in Pregnancy (IADPSG) and the World Health Organization (WHO) define GDM as fasting glucose ≥5.1 mmol/l or 1-h glucose post-OGTT of ≥10.0 mmol/ or 2-h glucose post-OGTT ≥8.5mmol/l [1, 2].

GDM is common, and although the reported prevalence varies considerably [3], the Irish ATLANTIC Diabetes in Pregnancy (DIP) study group found a prevalence of 12.4% using universal screening and IADPSG criteria within a regional population [4]. GDM is associated with several adverse pregnancy outcomes, including caesarean delivery, large (LGA) and small (SGA) for gestational age infants and neonatal intensive care unit (NICU) admission [5, 6]. In the longer term, women with GDM have an increased risk of type 2 diabetes and cardiovascular disease; increased rates of obesity, hyperglycaemic disorders and autism are noted in the offspring [6, 7]. The cost of diagnosing and managing GDM in Ireland is substantial with pregnancies affected by GDM incurring an additional cost of circa 30%, driven mainly by NICU admissions and caesarean delivery [8].

While treatment of GDM is associated with improved perinatal outcomes [9–11], the optimal approach to management is uncertain. Current guidelines recommend an initial period of medical nutritional therapy (MNT) with exercise and subsequent introduction of pharmacotherapy if adequate glycaemic control is not obtained. While this approach avoids pharmacotherapy in 60%, it results in hyperglycaemia in the other 40% of patients [12].

When glucose targets are not achieved, insulin therapy is typically prescribed, which is effective in normalising several perinatal outcomes to that of women with normal glucose tolerance (NGT), but increases caesarean delivery rates and the need for NICU care for their infants [13]. Insulin therapy also necessitates self-injection and is associated with an increased risk of maternal hypoglycaemia and excessive maternal gestational weight gain (GWG) [11, 14]. Excessive maternal weight gain is gaining momentum as an additional independent risk factor for macrosomia and LGA [11, 15]. Analysis of women from the ATLANTIC DIP cohort identified excessive GWG occurred in >60% of women with GDM [14]. This excessive GWG defined according to maternal body mass index (BMI) by the Institute of Medicine [16] independently increased the adjusted odds ratio (aOR) of LGA (aOR 2.0) and macrosomia (aOR 2.2) following adjustment for additional contributing variables. Treatment with insulin further increased the odds for LGA (aOR 2.8). These findings suggest that a focus on minimising excessive GWG is important and opens the debate regarding the usefulness and effectiveness of insulin as the preferred first-line treatment modality in women where MNT fails.

An alternate approach is using oral pharmacological therapies, with most experience using glyburide and metformin, introduced when MNT fails. A recent meta-analysis reported metformin to be better than insulin for perinatal outcomes, with glyburide inferior to insulin and metformin due to an increased risk of adverse perinatal outcomes [17–20]. Although metformin crosses the placenta, there is a body of evidence supporting its safety in pregnancy [21]. The most conclusive evidence comes from the metformin versus insulin for the treatment of gestational diabetes (MiG) trial where 752 women were randomised to metformin or insulin when MNT failed, which reported no increased risk of perinatal morbidity with metformin, compared to insulin. However, the trial was limited to obese patients who had failed MNT intervention [19].

Studies on the long-term effects of metformin are also encouraging. This includes data from mothers receiving metformin for polycystic ovarian syndrome (PCOS) where infants had normal weight height and social and motor skills at 18 months compared to unexposed infants [20]. In addition, longer term follow-up in the MiG trial reported no difference in total body fat of children at 2 years old between those whose mothers were treated with metformin or insulin [22].

Despite these safety data from select populations of pregnant women, evidence to support its use in a broader spectrum of all pregnancies affected by GDM is lacking, and no clinical trials have evaluated initiation of metformin at the time of GDM diagnosis, in tandem with MNT rather than when MNT has failed to control hyperglycaemia. In addition, there is no placebo-controlled trial of metformin in women with GDM. Based on the trials and observational data thus far, it is reasonable to believe that routine use of metformin in all women with GDM might reduce adverse maternal and neonatal outcomes across the spectrum of women affected.

### Objectives

The overall objective of the EMERGE trial is to determine if, in women with GDM managed with usual care, the early introduction of metformin vs. placebo reduces (a) the need for insulin use or hyperglycaemia (primary outcome); (b) excessive maternal weight gain; (c) maternal and neonatal morbidities; and (d) cost of treatment.

This protocol was written in adherence with the Standard Protocol Items: Recommendations for Interventional Trials (SPIRIT) Guidelines 2013 [23].

### Trial design

EMERGE is a phase III, parallel, superiority, randomised, double-blind, placebo-controlled trial of metformin (in addition to usual care) in women with GDM followed until delivery. The placebo was identical in taste, smell, appearance and packing to metformin.

## Methods: participants, interventions and outcomes

### Study setting

This study will take place across two sites—one tertiary referral centre/university and one smaller hospital with an average annual birth rate of 2800 and 1600 respectively. The geographical area and study population include women from urban and rural locations and women in both public and private health care.

### Eligibility criteria

The eligible population for the trial is pregnant women between the ages of 18 and 50 years with a diagnosis of GDM up to 28 weeks’ gestation (+ 6 days) diagnosed using a 75-g oral glucose tolerance test (OGTT) and WHO 2013 (IADPSG) criteria. Eligible women must be resident and intend to give birth within the selected trial sites, which include one tertiary referral centre/university and one smaller hospital with an average annual birth rate of 2800 and 1600 respectively. The geographical area and study population include women from urban and rural locations and women in both public and private health care.

To be eligible for the trial, women must meet the following eligibility criteria (item 10).

Inclusion criteria:

- Willing and able to provide written informed consent
- Women aged 18–50 years
- Pregnancy gestation up to 28 weeks (+ 6 days) confirmed by a positive pregnancy test
- Singleton pregnancy
- A diagnosis of GDM from a 75g OGTT according to WHO 2013 (IADPSG) criteria if any one of the following is achieved:
  - **◦** Fasting glucose **≥** 5.1mmol/l and <7mmol/l, or
  - **◦** One-hour post glucose load of **≥** 10mmol/2-hour
  - **◦** Two-hour post glucose load of **≥** 8.5 mmol/l and <11.1mmol/l
- Resident in the locality and intending to deliver within the trial site

Exclusion criteria:

- Women who have an established diagnosis of diabetes (type 1, type 2, monogenic or secondary)
- Women with fasting glucose ≥ 7mmol/l or a 2h value ≥ 11.1 mmol/l
- Multiple pregnancy (twins, triplets, etc.)
- Known intolerance to metformin
- Known contraindication to the use of metformin which includes
  - **◦** Renal insufficiency (defined as serum creatinine of greater than 130 μmol/L or creatinine clearance <60 ml/min)
  - **◦** Moderate to severe liver dysfunction (aspartate aminotransferase (AST) or alanine aminotransferase (ALT) greater than three times the upper limit of normal)
- Shock or sepsis at the time of recruitment
- Previous hypersensitivity to metformin
- Known foetal anomaly
- Known small for gestational age (foetal growth <10th percentile)
- Known current gestational hypertension, pre-eclampsia or ruptured membranes
- Subjects who have a history of drug or alcohol use that, in the opinion of the investigator, would interfere with adherence to study requirements
- Women with significant gastrointestinal problems such as severe vomiting, Crohn’s disease and colitis which will inadvertently affect the absorption of the study drug
- Women with congestive heart failure or a history of congestive heart failure
- Women with serious mental illness which would affect adherence to study medication or compliance with study protocol in the opinion of the investigator
- Women with rare hereditary problems of galactose intolerance, Lapp lactase deficiency or glucose-galactose malabsorption.

### Informed consent

Women who are willing to participate are enrolled by one of the study investigators who will obtain written consent. Enrolment is completed by a trained study coordinator. As the consent leaflets are written in English, a translator service provides for any participant who requires consent. For any participant who is unable to sign the consent form (due to visual of physical impairment) an impartial witness can sign on their behalf once verbal consent is obtained. A copy of the consent form is attached as Additional file 1. On the consent form, participants will be asked if they agree to use of their data should they choose to withdraw from the trial. Participants will also be asked for permission for the research team to share relevant data with people from the Universities taking part in the research or from regulatory authorities, where relevant. This trial does involve collecting biological specimens for ancillary studies.

## Interventions

### Choice of comparator

The compactor in this trial was placebo. The placebo was identical in taste, smell, appearance and packing to metformin.

### Intervention description

**Fig. 1:**
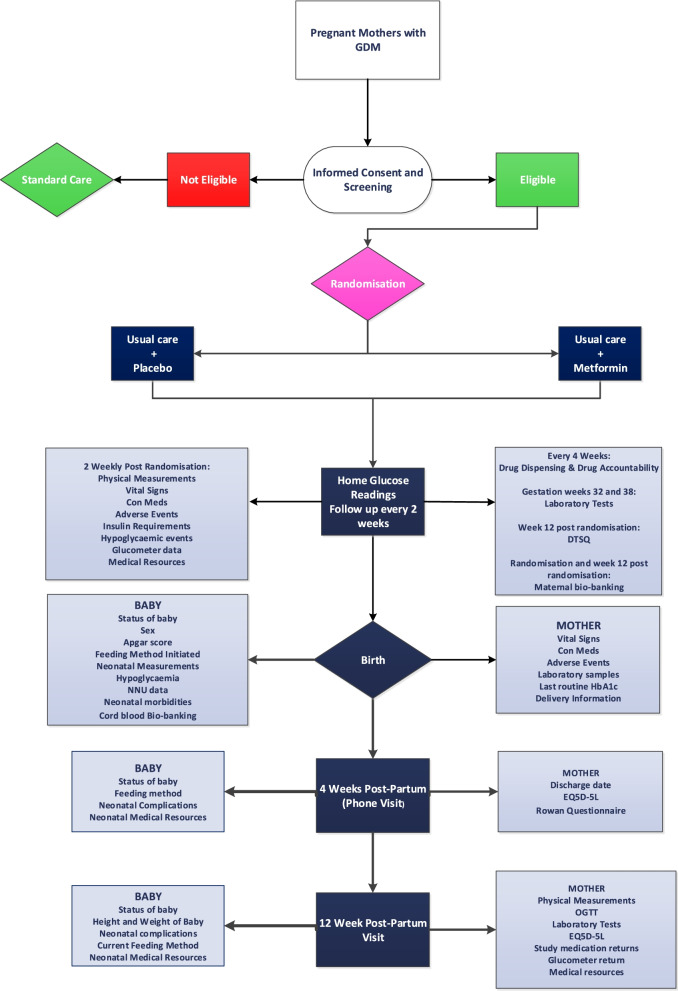
Schematic diagram of trial design

Eligible women are randomised into one of two groups: treatment group or placebo group. The treatment group receives metformin 500mg daily, with the dose titrated upwards every 2 days over 10 days, increasing to a maximum of 2500-mg metformin daily (5 tablets) or maximum tolerated dose, in addition to usual care (exercise and MNT), and taken until delivery. Women randomised to the placebo group receive one placebo tablet daily, with the dose titrated upwards every 2 days over 10 days, increasing to a maximum of five placebo tablets daily, in addition to usual care (exercise and MNT) and taken until delivery. Women are followed up at 4 and 12 weeks post-partum for other maternal and neonatal outcomes. The treatment and metformin group receive usual care, including MNT and information on exercise provided by the Diabetes team or trained delegate. The Diabetes team or trained delegate instruct women on using a glucometer, and the women perform 7-point glucose testing before and 1 h after meals and before bed. Women are supported as required by telephone contact from the Diabetes team or trained delegate throughout gestation and attend at 2–4 weekly intervals at an antenatal/diabetes clinic. Insulin may be commenced in women in each group as per normal practice if two or more home glucose readings are outside the pre-specified glucose targets (fasting ≤ 5mmol/L, 1 h postprandial ≤ 7mmol/L) (without reason) despite maximum oral therapy and MNT at any clinic visit. If insulin is initiated, metformin or placebo tablets are also continued at the maximum tolerated dose. Figure 1 outlines the overall trial design.

### Criteria for discontinuing of modifying allocated intervention

Reasons for drug discontinuation include intolerable side effects that do not respond to a dose reduction, an increase in their ALT or GGT to three times the upper limit of normal or a significant change in renal function as determined by the treating clinician.

All participants will also have their most recent ultrasound scan reviewed; any participant whose abdominal or foetal weight centiles drop below the 10th centile may have the investigational medicinal product interrupted if deemed necessary by the PI.

The dose of medication will be reduced in the event of troubling side effects; however, the dose will not be reduced in response to good glucose control, as glucose levels typically rise throughout pregnancy.

### Strategies to improve adherence to intervention

To promote retention, participants are offered several options to reduce the burden of multiple visits. The majority of visits are offered on the same day as schedule ante-natal care. Participants will also be offered phone call reviews if they cannot attend in person for an individual visit; medications will be transported to participants if needed using an approved courier service. A protocol deviation form will be completed in case of a protocol deviation, and a participant will be reviewed at the earliest possible opportunity.

### Relevant concomitant care permitted or prohibited during the trial

During the study, the use of other oral-hypoglycaemic agents is prohibited. We also asked participants to avoid the use of herbal remedies, the ingredients of which cannot be verified. The use of prescription medication (aside from oral-hypoglycaemic agents) is permitted including anti-hypertensives, anti-emetics, analgesia and proton pump inhibitors and use was recorded.

### Provisions for post-trial care

The sponsor and insurer provides indemnity cover to participants for the duration of their participation in the study and up until their 12-week post-partum follow-up. After that, their care will be returned to their own clinician.

## Outcomes

### Primary outcome measure

A composite primary outcome of insulin initiation or fasting venous glucose ≥ 5.1 mmol/L (on study-specific fasting laboratory glucose at gestational weeks 32 or 38) is used. This approach allows us to measure ‘treatment failure’ in two discreet ways. The introduction of insulin reflects clinically meaningful hyperglycaemia and is measured at any time during the clinical trial. In addition standardised fasting glucose is completed at gestational weeks 32 or 38 to capture additional participants who have fasting hyperglycaemia but have not had insulin introduced during the clinical trial.

### Secondary outcome measures

- Time to insulin initiation and insulin dose required
- Maternal morbidity at delivery (hypertensive disorders, antepartum and postpartum haemorrhage)
- Mode and time of delivery
- Postpartum glucose status, insulin resistance, and metabolic syndrome
- Postpartum body mass index (BMI), gestational weight gain (weight gain from booking visit to the last trial visit before delivery) and waist circumference
- Infant birth weight
- Neonatal height and head circumference at delivery
- Neonatal morbidities (need for neonatal care unit, respiratory distress, jaundice, congenital anomalies, Apgar score)
- Neonatal hypoglycaemia <2.6 mmol/L
- Cost-effectiveness of metformin treatment in addition to usual care
- Treatment acceptability to participants.
- Quality of life determined by EQ5D-5L questionnaire (a quality of life assessment with demonstrated reliability and validity in diabetes [24])

### Health economics analysis

A trial-based economic evaluation incorporating cost-utility analysis will be conducted to compare the alternative treatment strategies: (1) metformin and standard care for GDM and (2) standard care for GDM. Evidence collected on resource use and clinical outcome measures alongside the trial will provide the basis for the analysis over the trial follow-up period. A healthcare provider perspective will be adopted for costing. This will reflect the healthcare resources consumed in operating both treatment strategies, including health professional time input, diagnostic testing, dietary, exercise and prescription medications, consumables and materials, equipment and overheads. In addition, healthcare resource use for both treatment arms is collected. Unit costs will be applied to value resource use data and calculate care costs. For the cost-utility analysis, data collected using the EQ-5D-5L and SF-6D instruments will be used to generate quality-adjusted life-years (QALYs), which is the preferred outcome measure for economic evaluation [25]. An incremental analysis will be undertaken to compare the metformin plus standard GDM care intervention relative to the standard GDM care alternative. Univariate, multivariate and probabilistic sensitivity analyses will be employed to address uncertainty.

### Participant timeline

#### Screening for GDM and randomisation visit

Women receive a 75-g OGTT as part of routine clinical care. GDM is diagnosed according to the WHO 2013 (IADPSG) criteria if any one of the following is achieved: fasting glucose **≥** 5.1mmol/L and <7mmol/L, 1-h post glucose load of **≥** 10 mmol/L, and 2-h post glucose load of **≥** 8.5 mmol/L and <11.1mmol/L.

Those with a positive OGTT receive usual care of MNT and exercise advice from the Diabetes team or trained delegate and are approached for screening into the trial. Consenting women are screened for eligibility.

Screening consists of the following procedures:

- **◦** Review of inclusion/exclusion criteria
- **◦** Review of medical history, including previous pregnancy history
- **◦** Review of concomitant medications
- **◦** Current pregnancy information including the date of last menstrual period, estimated delivery date, gestational week, parity and gravida

Trial screening procedures are documented in the medical notes by the research nurse. The results of the screening visit are reviewed and eligibility is confirmed by an investigator before participants are assigned a trial-specific identification number and allocated to a study arm, through the IWRS. Randomisation occurs on the day of screening or up to 7 days post-screening. Once randomised, the following data are collected:

- **◦** Physical measurements (heart rate, systolic and diastolic blood pressure, height, weight and body mass index (BMI))
- **◦** Demographics (date of birth, ethnicity)
- **◦** Social history (smoking and alcohol)
- **◦** Socioeconomic status
- **◦** Gastrointestinal symptoms
- **◦** EuroQol five-dimension measurement tool (EQ5D-5L) questionnaire (a standardised measure of health status which provides a measure of health for clinical and economic appraisal) [26]
- **◦** Laboratory tests (HbA1C, insulin, C peptide, total cholesterol, HDL cholesterol, LDL cholesterol triglycerides, urea, creatinine, alanine aminotransferase, aspartate transaminase)
- **◦** Healthcare resources used since diagnosis of pregnancy
- **◦** Usual care received

Once randomisation has occurred, study medication is dispensed (in line with drug accountability practice) and participants are given administration instructions.

#### Prenatal visit and procedures

Prenatal visits occur approximately every 2–4 weeks post-randomisation, in line with routine antenatal clinic visits. The following data is collected by trained study coordinators and procedures performed:

- Physical measurements (heart rate, systolic and diastolic blood pressure, weight and BMI)
- Gestational age in weeks
- Review of concomitant medications
- Review of insulin requirements
- Review of adverse events
- Gastrointestinal symptom review
- Review of hypoglycaemic events
- Review of medical resources used
- Glucometer data download

In addition, the following data is collected at specified time points**:**

- Study drug dispensing (every four4 weeks).
- Usual care received (week 4).
  - **◦** Laboratory tests (HbA1C, fasting glucose, total cholesterol, HDL cholesterol, LDL cholesterol, triglycerides, urea, creatinine, alanine aminotransferase, aspartate transaminase) at gestational weeks 32 (± 1 week) and 38 (± week)).
- Diabetes Treatment Satisfaction Questionnaire (DTSQ) (week 12 only)—DTSQ is a validity treatment satisfaction assessment tool [27].
- Study Drug Accountability (every 4 weeks)—participants will be asked to bring their medications with them to each visit and compliance will be calculated from the number of tablets taken.

#### Birth visit

The birth visit occurs within 72 h after birth while the woman is in the postnatal ward. Should the woman be discharged early, or it is not possible to conduct the visit within the window (e.g. delivery occurs out of hours), every effort is made to gather the information from medical notes or through telephone contact with the participant. The following data are collected and procedures performed:

- Vital signs (heart rate, systolic and diastolic blood pressure)
- Review of concomitant medications
- Review of adverse events
- Last routine HbA1c recorded
- Delivery information (time, date and mode of delivery and complications)
- Feeding method initiated
- Neonatal procedures (status of baby, sex, neonatal measurements, Apgar score, hypoglycaemia, respiratory distress, jaundice and congenital anomalies)
- Neonatal care unit data
- Neonatal medical resources

A cord blood sample is collected if the participant provided informed consent for this sub-study.

#### Phone visit (visit 1 post-partum)

A phone visit occurs 4 weeks (± 5 days) post-partum. The following data is collected:

- Status of the baby
- Current feeding method
- Neonatal complications
- Discharge date
- EQ5D-5L and Rowan Questionnaires

An appointment is scheduled for 12-week post-partum.

#### Twelve-week post-partum visit (visit 2 post-partum)

The post-partum visit occurs 12 weeks post-partum (± 4 weeks) and is conducted in person. The following data is collected and procedures performed:

- Physical measurements (heart rate, systolic and diastolic blood pressure, height, weight and BMI, waist circumference)
- Seventy-five-gramme OGTT with samples fasting and 1-h and 2-h post glucose lead
- Laboratory tests to include fasting insulin and C peptide, HbA1C, total cholesterol, HDL cholesterol, LDL cholesterol and triglycerides
- EQ5D-5L Questionnaire
- Study medication returns
- Status of baby
- Neonatal complications
- Current feeding method
- Return Glucometer and data download
- Adverse events (mother and baby)
- Medical care received since delivery

**Fig. 2:**
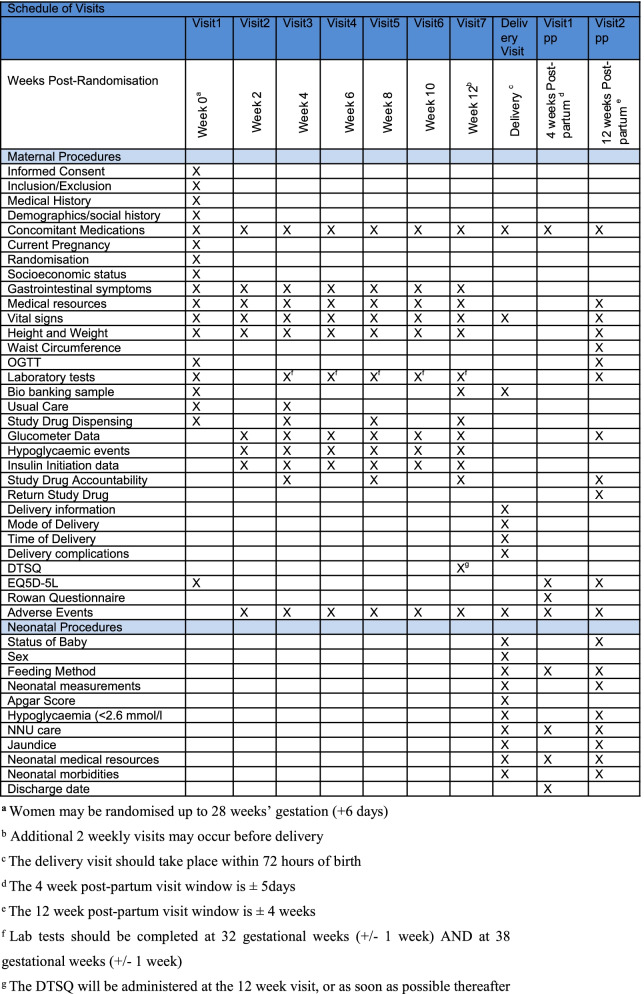
Schedule of Visits and Procedures for the EMERGE Trial. ^**a**^Women may be randomised up to 28 weeks’ gestation (+6 days). ^b^Additional 2 weekly visits may occur before delivery. ^c^The delivery visit should take place within 72 h of birth. ^d^The 4-week post-partum visit window is ± 5days. ^e^The 12-week post-partum visit window is ± 4 weeks. ^f^Lab tests should be completed at 32 gestational weeks (± 1 week) AND at 38 gestational weeks (± 1 week). ^g^The DTSQ will be administered at the 12-week visit, or as soon as possible thereafter

An outline of scheduled study assessments and procedures is presented in Fig. 2.

#### Sample size

Our sample size is based on the following assumptions:

- Forty percent of participants in the placebo group will require insulin, based on the MiG trial [19] and local unpublished data from University Hospital Galway
- Ability to detect a minimum of 30% relative risk reduction (RRR) in the proportion of women requiring insulin in the experimental (metformin) group (40 to 28% absolute reduction)
- A significance level of 0.05 and 80% power
- A dropout rate of 5% or less and
- Non-adherence rate of 8% in the metformin group

Based on these assumptions, we require an overall sample size of 550 participants. This sample size will also have 80% power to demonstrate a difference between the proportions of 12% or more (i.e. a reduction from 60 to 48%) in the secondary outcome of excessive gestational weight gain. There is a greater concern with loss to follow-up for the secondary outcome of baseline to post-partum weight change, as post-pregnancy follow-up rates have been reported in some studies to be less than 70%. For this outcome, even with a loss to follow-up of 50%, the resulting 138 per arm will have 80% power, at the 0.05 significance level, to detect a minimum difference in mean weight change of 1.36kgs (assuming a standard deviation of the change in weight of 4kgs). However, every effort will be made to achieve follow-up rates of >95% for post-partum follow-up. We will implement several strategies to enhance follow-up for this outcome (e.g. home monitoring of weight).

#### Recruitment

Women are recruited from antenatal clinics in both participating hospitals. At their first visit following GDM diagnosis, women receive a standard lifestyle and education intervention. Eligible women are given an information leaflet inviting them to participate and receive a phone between 7 and 14 days later.

## Assignment of interventions: allocation

### Sequence generation

A minimisation strategy allows equal numbers of women with a BMI ≤/>30 and a history of GDM to be distributed between groups. A fixed block size of 4 are used to ensure similar numbers of women in each intervention arm throughout the trial and equal numbers in each arm by the end of the study.

### Concealment mechanism

A secure, interactive web-based randomisation system (IWRS) is used to randomly assign participants to metformin or placebo with a 1:1 allocation ratio.

### Implementation

Participating sites obtain allocated treatment numbers and subject IDs after confirming eligibility. This centralised system ensures allocation concealment, preventing trial staff from knowing which treatment group is allocated.

### Blinding

This trial is conducted in a double-blind fashion with a placebo, identical to metformin tablets, to minimise performance and detection biases. Site investigators, site personnel, participants and outcome assessors are blinded to treatment allocation. In the case of an emergency, when knowledge of the participant’s study treatment assignment is essential for clinical management, an investigator may un-blind a participant. Any intentional or unintentional breaking of the blind is recorded and reported to the sponsor as soon as possible.

## Data collection and management

### Plans for assessment and collection of outcomes

Source documents for the trial include hospital records, procedure reports and data collection forms stored safely to maximise confidentiality. These documents are used to record data onto study-specific, pseudo-anonymised and password-protected case report forms (CRF), where the participant is referred to by a study-specific number (allocated at study enrolment) and initials. CRF data is entered into a secure, study-specific, electronic database within a clinical data management system (CDMS) maintained by the trial Sponsor. The CDMS has an auto-alert system for biometric reference ranges (for example, the research assistant entering a systolic blood pressure of 200mmHg will be asked to confirm this is correct). The data manager, and database developer, maintains a study-specific data management plan, including a detailed data management file stored by the trial Sponsor.

### Plans to promote participant retention and complete follow-up

To promote retention, participants are offered several options to reduce the burden of multiple visits. The majority of visits are offered on the same day as schedule ante-natal care. Participants will also be offered phone call reviews if they cannot attend in person for an individual visit; medications will be transported to participants if needed using an approved courier service. A protocol deviation form will be completed in case of a protocol deviation, and a participant will be reviewed at the earliest possible opportunity.

### Confidentiality

Source documents for the trial include hospital records, procedure reports and data collection forms stored safely to maximise confidentiality. These documents are used to record data onto study-specific, pseudo-anonymised and password-protected case report forms (CRF), where the participant is referred to by a study-specific number (allocated at study enrolment) and initials. CRF data is entered into a secure, study-specific, electronic database within a clinical data management system (CDMS) maintained by the trial Sponsor.

### Plans for collection, laboratory evaluation and storage of biological specimens for genetic or molecular analysis in this/future studies

Blood samples will be taken as per the above schedule. The lab in Galway University Hospital will analyse these samples.

## Statistical methods

### Statistical methods for primary and secondary outcomes

The intention-to-treat (ITT) analysis set will include all randomised participants. The safety analysis set (SAS) will include all randomised participants who received at least one dose of study medication. The study population demographic and baseline characteristics will be summarised using graphical displays and descriptive statistics for each treatment group. Suitable numerical and graphical techniques will compare the primary and secondary responses and the balance in explanatory variables at baseline. The primary analysis will be a two-sample comparison of the reduction in the proportion of women needing insulin between treatment and control arms using an exact test for a binomial response. We will also conduct a logistic regression analysis to adjust for differences in baseline covariates between treatment groups. Several strategies including explanatory variables will be employed where penalisation for multi-collinearity will be achieved using ridge penalties. Following this, the most parsimonious subset of predictor variables will be identified using computationally intensive data-driven techniques such as the classification trees and the Lasso penalty. A secondary exploratory analysis will involve comparing the time to insulin initiation between the treatment groups, initially using the log-rank test and then the proportional hazards model to adjust for patient characteristics as appropriate. Repeated measures ANOVA will be used to evaluate the effect of the intervention on the secondary outcome of mean change in weight (from baseline to post-partum follow-up).

The primary efficacy outcome is a composite of

- Insulin initiation at any point up to delivery (Yes/No)
- Fasting glucose value ≥ 5.1 mmol/l at either week 32 or 38 of gestation

Secondary efficacy outcomes include:

- Maternal BMI, waist circumference and maternal gestational weight gain
- Blood glucose status, insulin resistance status and metabolic syndrome postpartum
- The proportion of infants with morbidities (need for neonatal unit care, respiratory distress, jaundice, congenital anomalies, Apgar score <7 at five minutess after birth, neonatal hypoglycaemia, 2.6mmol/L on one or more occasions within 30–60 min after birth)
- Infant birth weight
- Proportion of maternal morbidities

Health economic outcomes include:

- EQ5D-5L
- Quality-adjusted life years (QALYs)
- Costs of healthcare associated with the intervention and control arms

The level of statistical significance will be set at alpha of 0.05 for all analyses, i.e. a *p-*value <0.05 with 95% CIs not containing zero will be considered statistically significant.

### Interim analysis

No interim analysis is planned.

### Methods for additional analyses

Pre-specified subgroup analyses are planned by age, prior history of GDM, week of gestation recruited and maternal weight, BMI and waist-hip ratio.

### Methods in analysis to handle protocol non-adherence and missing data

An analysis of missing data for all relevant outcomes will be carried out to identify the likely missing data mechanism and to describe the pattern of missingness. If the potential impact of missing data is non-negligible (moderate to high proportion of missing data and/or potential for bias), a suitable multiple imputation strategy will then be employed to determine the sensitivity of missing data on the inference gleaned from the analysis of the primary and secondary outcomes.

### Plans to give access to the full protocol, participant-level data and statistical code

We plan to grant public access to the complete protocol; however, we will not allow access to participant-level dataset and or statistical code.

## Oversight and monitoring

### Composition of the coordinating centre and trial steering committee

The National University of Ireland, Galway (NUI Galway) is the Sponsor of the EMERGE trial. The Chief Investigator (CI) has overall responsibility for the conduct of the trial. The sponsor maintains clinical trial insurance coverage for the trial per Irish laws and regulations. The State Claims Agency Clinical Indemnity Scheme provides clinical indemnity for any harm caused to patients by the design of the research protocol. Additionally, indemnity to allow for no-fault compensation is provided for by NUI Galway. The agreements put in place between the Sponsor and individual participating sites cover the indemnity provision for negligent harm. This indemnity covers participants for the duration of their participation in the study and up until their 12-week post-partum follow-up. After that, their care will be returned to their own clinician. The trial is funded by the Health Research Board (HRB) and conducted following the ethical principles that have their origin in the Declaration of Helsinki. The protocol is approved by a recognised Research Ethic Committees (REC) for all participating sites.

The study sponsor and funder had no role in the study design; collection, management, analysis, and interpretation of data; writing of the report; or the decision to submit the report for publication.

Source documents for the trial include hospital records, procedure reports and data collection forms stored safely to maximise confidentiality. These documents are used to record data onto study-specific, pseudo-anonymised and password-protected case report forms (CRF), where the participant is referred to by a study-specific number (allocated at study enrolment) and initials. CRF data is entered into a secure, study-specific, electronic database within a clinical data management system (CDMS) maintained by the trial Sponsor.

### Composition of the data monitoring committee, its role and reporting structure

The Data Safety Monitoring Committee (DSMC) is composed of an obstetrician, a midwife, an endocrinologist and two statisticians. The obstetrician will act as chair and facilitate and summarise discussions and all members are independent of the trial.

The primary responsibilities of the DSMC are:

1. To make written recommendations to the trial steering committee (TSC) concerning the continuation, modification, or termination of the trial.
2. Consider any requests for release of interim trial data and make recommendations to the TSC on the advisability of this.
3. Review major proposed modifications to the study prior to their implementation (e.g. termination, increasing target sample size).
4. Maintain confidentiality during all phases of DSMB review and deliberations.
5. Review SAEs and suspected unexpected serious adverse reactions (SUSARs) as appropriate

The DSMC have visibility of all adverse events and serious adverse events and have the authority to terminate the trial at any point based on safety concerns. The DSMC will report their recommendations and decisions to the TSC or sponsor’s representative by a written communication (letter or email) email, usually within 3 weeks of the meeting. Further details are available in the DSMC Charter version 4.0 (6 September 2021).

### Adverse event reporting and harms

A review of any adverse events will take place at each prenatal visit. The principal investigator (PI) or co-PI will grade adverse events as mild or moderate by the principal investigator (PI) or co-PI. Any event which results in hospitalisation/prolongation of hospitalisation/a medically important event/requires intervention to prevent permanent impairment/birth defect/disability/permanent damage or death will be graded as a serious adverse event (SAE). Any spontaneously reported adverse event will be recorded and graded at the time of reporting.

### Auditing

Trial monitoring visits are carried out according to the trial monitoring plan and are independent from the trial investigators.

### Plans for communicating important protocol amendments to relevant parties (e.g. trial participants, ethical committees)

Any modifications to the protocol will require the approval of the sponsor and ethics committee at each centre. Formal amendments will be made and each centre will be given 30 days to consider changes before approving for said amendments.

Changes include alterations to inclusion or exclusion criteria, primary or secondary outcomes, study size or conduct of the study.

Once changes have been agreed on and approved each centre will be sent written notice of this change in protocol and key personnel will be asked to signify by return email that the amendments are noted and understood. The updated protocol should be in the site file at all times.

Minor changes, e.g. spelling or administrative modifications considered minor and that do not affect the conduct of the study, will be agreed upon by the trial coordinators and sponsor and the ethics committee will be notified of any changes at the end of each month

.

### Dissemination

The PI, biostatistician and sponsor will have access to the final dataset. There are no contractual agreements to limit investigator access.

A report will be completed for the funding bodies, and the trial outcomes will be written for peer-reviewed publications and disseminated to international lay and scientific audiences. The participants will be informed of the results and their allocation via personalised correspondence. There are no publication restrictions.

Only contributors who contribute substantially to the formation of the protocol and conduct of the trial will be considered eligible for authorship. Professional writers will not be used.

*Sponsorship, indemnity, finance and ethics*

## Discussion

Data to support the routine use of metformin in relatively unselected populations of pregnancies affected by GDM are lacking, as is a placebo-controlled trial in a GDM population. The EMERGE trial will determine whether early routine use of metformin reduces the need for insulin use or hyperglycaemia and reduces excessive maternal weight gain, maternal and neonatal morbidities and the cost of treatment for women with GDM.

## Conclusion

The EMERGE trial aims to provide robust evidence for the efficacy of metformin in women with GDM diagnosed using the WHO 2013 (IADPSG) criteria. It will inform patients, caregivers and funding agencies regarding the use of metformin in GDM and has the potential to change practice.

### Trial status

EMERGE protocol version 8 (27 July 2021) is currently in use. Recruitment began on June 15, 2017, and is expected to be complete on September 30, 2022.

## Supplementary Information

**Additional file 1.**

## Data Availability

We plan to grant public access to the full protocol; however, we will not allow access to participant-level dataset and or statistical code.

## Abbreviations

GDM: Gestational diabetes mellitus
MNT: Medical nutritional therapy
BMI: Body mass index
EQ5D-5L: EuroQol five**-**dimension measurement tool
IADSPG: International Association for the Study of Diabetes in Pregnancy
DIP: Diabetes in pregnancy
LGA: Large for gestational age
SGA: Small for gestation age
NICU: Neonatal intensive care unit
NGT: Normal glucose tolerance
GWG: Gestational weight gain
aOR: Adjusted odds ratio
PCOS: Polycystic ovarian syndrome
OGTT: Oral glucose tolerance test
AST: Aspartate aminotransferase
ALT: Alanine aminotransferase
IWRS: Interactive web-based randomisation system
EQD5D-5L: Euroqol five dimensions measurement tool
PI: Principal Investigator
DTSQ: Diabetes Treatment Satisfaction Questionnaire
RRR: Relative risk reduction
ITT: Intention-to-treat
SAS: Safety analysis set
QALYs: Quality-adjusted life years
CRF: Case report forms
CDMS: Clinical data management system
HRB: Health Research Board
REC: Research Ethics Committees
TSC: Trial Steering Committee
SUSAR: Suspected unexpected serious adverse reaction
