# Supplementary material for: A randomised placebo-controlled trial of the effectiveness of early metformin in addition to usual care in the reduction of gestational diabetes mellitus effects (EMERGE): study protocol
Source: Trials. 2022 Sep 21;23:795. doi: 10.1186/s13063-022-06694-y (PMC9494837; doi:10.1186/s13063-022-06694-y)
Supplement: Supplementary file 1 — Additional file 1. [file 13063_2022_6694_MOESM1_ESM.pdf]

Participant Information Leaflet: EMERGE Trial

Study Number: NUIG-2016-01

Study Title: A randomised placebo controlled trial of the effectiveness of early metformin in addition to usual care in the treatment of Gestational Diabetes Mellitus (GDM)

Principal Investigator: Prof. Fidelma Dunne

Introduction

You are invited to take part in a study on gestational diabetes mellitus (GDM). GDM is diagnosed when blood sugar levels are high during pregnancy. You have been chosen to take part in this trial because the oral glucose tolerance test (OGTT) you had was positive. This means that your blood sugars are above the recommended level in pregnancy.

Taking part in this study is voluntary, whether or not you take part is your decision. If you don't want to take part, you don't have to give a reason, and it won't affect the care you receive. If you do want to take part now, but change your mind later, you can withdraw from the study at any time.

This Participant Information Leaflet will help you decide if you'd like to take part. It sets out why we are doing the study, what your participation would involve, what the benefits and risks to you might be, and what would happen after the study ends. We will go through this information with you and answer any questions you may have. You do not have to decide today whether or not you will participate in this study. Before you decide you may want to talk about the study with other people, such as family, friends, or healthcare providers. Feel free to do this.

If you agree to take part in the EMERGE study, you will be asked to sign the Consent Form on page 9 and 10 of this document. You will be given a copy of this Participant Information Sheet and the Consent Forms to keep for your own records.

What is the Purpose of the Study?

The main purpose of the study is to test whether a medication called metformin (commonly used to treat diabetes outside of pregnancy) is good at controlling blood sugar levels in women with high blood sugars during pregnancy.

Metformin is a medication that has been used for 30 years in treating diabetes and infertility. It has been used in Australia and New Zealand in pregnancy, and as of 2015, it has been recommended for use in the UK. Metformin has been shown to be as good as insulin in lowering blood sugars and safe for use during pregnancy in previous clinical trials in obese women. Metformin may also reduce the chance of women with GDM developing diabetes after pregnancy. We want to determine if metformin is beneficial for all women with GDM irrespective of their weight.

GDM affects approximately one in eight pregnant women in Ireland. It is associated with a higher risk of developing long-term diabetes after pregnancy, and pregnancy complications including high blood pressure, too much fluid around the baby and delivery by caesarean section. Infants also have a higher risk of being born overweight, having low blood sugars after delivery or jaundice or breathing difficulties that may require admission to the neonatal intensive care unit. Infants also have a higher risk of developing diabetes later in life.

If we can keep the blood sugar normal during the pregnancy we can reduce the risk of these events happening. The current treatment for GDM to keep blood sugars in the normal range is exercise (30 minutes per day) and changes to your diet. If blood sugars remain high despite these measures, women are treated with insulin by injection. Insulin is effective in keeping blood sugars at normal levels, but it has possible side effects for the mother (including excess weight gain, low blood sugars, and an increased risk of delivery by caesarean section), and the infant (including excess weight gain).

The study will be carried out in a minimum of two hospitals in Ireland and will include about 550 women. The study is funded by the Health Research Board and is being coordinated by the Clinical Research Facility in Galway.

The Ethics Committee that is responsible for overseeing research at your center has carefully reviewed and approved this study to make sure that your rights are protected.

What is a randomised placebo controlled trial

A randomised placebo controlled trial is a research study in which patients are allocated at random (by chance alone) to receive one of two clinical interventions. In this case, one of these interventions is active treatment with metformin, while the other is a ‘dummy’ treatment called placebo. All participants taking part in this study will receive usual standard of care for GDM.

What will my participation in the study involve?

<u>Screening & Randomisation visit</u>

If you decide to take part in this trial, you will be asked to sign the consent form on page 9 and 10 of this document. The study staff will then perform the following procedures:

- Check that you meet all of the eligibility criteria for the study
- Record information about your medical history, medications, demographics, and socioeconomic status
- Measure your blood pressure, heart rate, height and weight
- Administer a questionnaire about your quality of life
- Take some blood samples

All women participating in the study will receive our usual care of nutritional and exercise advice to control blood sugars. You will be taught how to use a device called a glucometer to check your blood sugar each day, and keep a diary to monitor your blood sugar levels.
Then, you will be randomly assigned to 1 of 2 treatments for the duration of the study (you will have a 1 in 2 chance of receiving one of the following):

Metformin 500mg up to 5 tablets daily + Usual Care (exercise & nutritional advice)	OR	Placebo tablet up to 5 tablets daily + Usual Care (exercise & nutritional advice)

The study staff will give you information on how to take these medications. Study medication will be taken up until the birth of your baby. During the study, you may or may not require insulin, as per usual care. Your doctor will prescribe insulin should you need it and provide you with instructions on how to administer it.

Neither you nor your study doctor will know which study medicines you are taking. In an emergency, your study doctor can find out what you are taking. At the end of the study, your study doctor will be told about the results of the study and which study medicines you were taking. He or she will discuss this information with you if you wish.

The screening and randomisation visit will take about 1 hour.

Follow Up Visits:

Follow-up visits will occur every two weeks until the birth of your baby. These follow up visits will occur either in person on the same day as your routine antenatal visits as much as possible or as a telephone visit.

At the in-person visits the study staff will do the following:

- Measure your blood pressure, heart rate and weight
- Record any changes to your medicines
- Check that you are taking the study medications
- Ask you about any medical resources you have used

You will have blood samples taken twice during this period (at 32 weeks and 38 weeks of pregnancy).

You will be asked to bring your glucometer to every study visit along with a diary to monitor your blood sugar readings. The data from the glucometer will be stored and analysed as part of a future observational study should you consent to this.

Each follow up visit will take about 25 minutes and will coincide with your routine antenatal visits as much as possible.

After Birth Visits:

Delivery: Once you have given birth to your baby, a study nurse will visit you in the hospital. At this visit the nurse will do the following:

- Measure your blood pressure and heart rate (we may also take these from your medical notes)
- Record any changes to your medicines
- Collect information about your babies' birth
- Collect information on your baby including sex, feeding method, and any complications
- Collect physical measurements of your baby including weight, height, head circumference, waist circumference and arm circumference

4 weeks after birth: You will be contacted by phone 4 weeks after birth by the study nurse to see how you and your baby are doing. You will be asked to complete a questionnaire over the phone. An appointment will also be scheduled for your final study visit.

12 weeks after birth: Your final visit will be approximately 12 weeks after birth, which is usual care for women with GDM. At this visit the study staff will do the following:

- Measure your blood pressure, heart rate, height, weight and waist circumference
- Blood tests to include an oral glucose tolerance test
- Complete a questionnaire
- Collect information about your baby including feeding method and status
- Record any changes to your medicines
- Ask you about any medical resources you have used since delivery

You will also be asked to return any study medications you have as well as the glucometer at this visit.

What are the possible benefits and risks of the study?
The study treatment may or may not be of personal benefit to you. Even if not of benefit to you, the results of this study may help in finding new treatments that lower the risk of complications in women with GDM.

The study may show that metformin is effective at controlling blood sugar levels in all women with GDM but without the side effects seen with insulin. It may reduce excessive weight gain for the mother and reduce risk of diabetes in the future for the mother. For the baby, metformin may show a reduction in excessive body fat.

Known risks of this study include the usual risks associated with taking blood (discomfort at the injection site, dizziness when having blood drawn) and possible side effects of study medicine. The most commonly reported side effects of metformin are nausea and vomiting, diarrhoea, and flatulence. Other very rare side effects of metformin include lactic acidosis (increased lactate in the blood) and decreased vitamin B12 levels usually associated with long term use. In addition to these, your study doctor has a document containing information on side effects that occur with less frequency and can discuss these with you.

Although the medicine being tested in this study has been used in many patients, unexpected side effects can still occur. You should review your symptoms (if any) with your study doctor.

Prohibited medications

Other oral anti diabetic medications cannot be taken for the duration of this study. It is very important that you tell your study doctor about any new medicines (prescription and non-prescription, herbal supplements and Chinese medicines) that you may start taking during the study.

Cost and Compensation for Participation in this Study

You will not be charged for the study medications or any of the tests and procedures performed. You will not be paid for your participation. If you require insulin during the study, this will be prescribed by your doctor; insulin and its cost are not provided by the study. Any reasonable costs incurred as a direct result of your participation (e.g. travel expenses) will be reimbursed on production of valid receipts.

Compensation for Injury Resulting from this Study

NUI Galway maintains insurance coverage for this study in accordance with Irish laws and regulations.

If you become ill or are physically injured as a result of participation in this study, please contact the study doctor immediately at 086 0208726 or 091 524222. He or she will treat you or refer you for treatment.

In no way does signing this consent form waive your legal rights or relieve the study doctor, sponsor or involved institutions from their legal and professional responsibilities.

Where can I get further information?

If you have any further questions about the study or if you want to opt out of the study, you can rest assured it won't affect the quality of treatment you get in the future.

If you need any further information now or at any time in the future, please contact your doctor; 091 524222 or your Clinical Research Nurse at:
Michelle Courcy Byrnes 086 0208726

Confidentiality and authorization to collect, use, and disclose personal information

Unless required by law, your name and personal information will not be given to anyone not involved in the study. Your personal information collected as part of this study, will be made available to study staff at your Centre.

Please be aware that the data about you that will be collected in the study includes special categories of personal data, namely information about:

- your age, sex and ethnic background
- your health and medical conditions including your past medical history
- your pregnancy and outcome of your pregnancy
- your treatments and your response to treatment
- your baby's health

If needed, the study staff may contact your personal physician to collect additional medical information, or a personal contact in the event that you cannot be reached. Your study information will be identified in the database by a code and not your name. The study staff will keep record of which code belongs to you.

The sponsor, ethics committees, and health authority inspectors, such as the Health Products Regulatory Authority, may visit the trial site and access you and your baby's study information. These people will use your personal information to check that the study is conducted correctly and to make sure the study information is accurate. These people are all required to keep the study information confidential by the nature of their work or by confidentiality agreements.

The Sponsor (NUI Galway) will keep any information they receive confidential as required by Irish and EU law. The study information will be used only for research purposes mentioned above. If the results of this study are published or presented in a meeting, you will not be named and nobody will be able to tell from the information provided that you were in the study.

You have the right to review your study information and medical records and request changes to the study information if it is not correct. However, please note that during the study, your access to study information may be limited because we do not want study staff to know what treatment you were assigned to, since this can affect the quality of the study. You may have access to the study information at the end of the study.

A description of this clinical trial will be available on http://clinicaltrials.gov , as required by Law. This Web site will not include information that can identify you. At most, the web site will include a summary of the results. You can search this web site at any time.

If you have any questions about the collection and use of yours or your baby's information or would like to exercise rights that you may have regarding this information, you should ask your study doctor.

Data Privacy and GDPR

What rights does GDPR provide?

Providing written informed consent means the CRFG can process your personal data for purposes stated in the clinical trial. The characteristics of the written informed consent process are in line with the requirements of Good Clinical Practice and Data Protection requirements ensuring your rights are protected. These include your right, at any time, to withdraw your consent from continuing in the clinical research study. Additionally, under GDPR, you have the following enhanced rights in relation to how we use your personal data:

For your data collected within the study you can apply the following privacy rights:

- Request information about the handling of your data. However, to protect the scientific integrity of the study you may not be able to receive access to some of the data before the study ends.
- Request correction of data about you if it is incorrect or incomplete. During the assessment of this request, you have the right to restrict the processing data about you.

- Request transfer of data about you to you or someone else in a commonly used format.
- File a complaint with a data protection authority.
- Withdraw your consent at any time without giving reason. You can withdraw your consent for the study treatment and/or further follow up, without withdrawing consent for handling your data. You may also withdraw consent to the handling of your data but please note previous data processing, before this, is legally covered by your original consent. After this withdrawal no further data will be collected from you.
- Along with your withdrawal, you have the right to request the deletion of data about you if your data is no longer needed or there is no other legal requirement for its use (e.g. to assess the study drug health authority, sponsor etc.) to ensure that your legitimate interests will not be compromised.

If you wish to apply any of your data privacy rights with respect to your data, please inform your study doctor.

Sharing your Data:
Data is processed during the EMERGE study in adherence to the processes outlined by the Data Controller. The Data Controllers of the EMERGE study are Prof. Fidelma Dunne and NUI Galway.

Sharing of your data by the CRFG is restricted. We only share your information as necessary in line with the requirements of the Study to facilitate the conduct of the research and to ensure your safety and protection.

Transfer of encoded data to other countries:
Your encoded study data may be transferred within and/or outside the EU in line with reporting requirements to the drug manufacturer, MERCK.

Retention of encoded data:
Your encoded data will be stored for at least 25 years after the end of the study, or longer, if needed for legal requirements.

During the course of the EMERGE study the data may be shared with:

- Individuals undertaking controller tasks required for the conduct of the study.
- Data processors who are delegated the task of managing the data by the Controller (in the case of EMERGE Data Manager(s) and Biostatisician (s) within the Data Management and Biostatistics Department of HRB Clinical Research Facility Galway, National University of Ireland, Galway University Hospital, Galway, H91 YR71, Ireland, Tel: +353 91 494369) are delegated the responsibility of Data processing.
- The Ethics committee(s) (National Office for Research Ethics Committees, Grattan House, 67-72 Lower Mount Street, Dublin 2, D02 H638 and/or Galway University Hospitals Research Ethics Committee, Merlin Park).
- The Data Safety Committee appointed for the EMERGE Study.
- The Competent Authority HPRA and other applicable Regulatory bodies as required to facilitate audit and inspection.

Where can I get further information on Data Protection and GDPR

- If you have any questions concerning any personal data you believe or know the organisation holds about you, please contact your appointed research nurse or the Chief Investigator of the study (Professor Fidelma Dunne at **091 495074** or the Clinical Research Nurse at 086 0208726.

- You can also contact **091 494369**

- You can also contact the following Data Protection Officers:
  - NUI Galway Data Protection Officer at dataprotection@nuigalway.ie

    and /or

  - HSE West Deputy Data Protection Officer in writing at ddpo.west@hse.ie or by telephone on 091 775819.

- In the event that you wish to make a complaint about how your personal data is being processed by us or how your complaint has been handled, you have the right to lodge a complaint directly with the supervisory authority:
  - Data Protection Commissioner: Office of the Data Protection Commissioner. Canal House, Station Road, Portarlington, Co. Laois, R32 AP23, Ireland. Phone +353 (0761) 104 800 | LoCall 1890 25 22 31 | Fax +353 57 868 4757 | email info@dataprotection.ie

For further detailed information regarding GDPR please refer to CRFG Website: http://www.nuigalway.ie/hrbcrfg

Who do I contact for more information or if I have concerns?
Please contact study staff at any time if you have questions or concerns about the study or if you have any injury, illness or side effects.

Study physician: 091 524222
Telephone number: 086 0208726
After office hours: 091 524222

If you have questions related to your rights as a research study participant, please contact the relevant Ethics Committee (National Office for Research Ethics Committees on (01) 234 5000 or Galway University Hospitals Clinical Research Ethics Committee on (091) 775 022).

EMERGE STUDY CONSENT FORM

Study Centre Name: Galway University Hospital
Study Doctor Name: Prof. Fidelma Dunne
Taking part in this study is voluntary however, to do so it is necessary for you to consent. Your consent is the legal basis for handling your data and without this we would not be able to use your data for the conduct and analysis of this study. You are not legally obliged to consent or to provide your data or your baby's data, but it is necessary if you wish to take part in the study:

By signing below, I agree to the following: Please initial each box

1. I confirm that I have read this document and had its contents explained to me. I understand the purpose of this study and what will happen to me in this study. I have had a chance to consider the information, discuss the study, and ask questions about the study which have been answered. ☐

2. I understand that my participation is voluntary and that I am free to withdraw at any time, without giving a reason, without my medical care or legal rights being affected. I understand that by signing this consent form, I am not waiving any legal rights that I otherwise have. ☐

3. I understand that data collected for use in the study will include my information and my baby's information. ☐

4. I understand that relevant sections of my medical notes and data collected during the study, may be looked at by responsible individuals, auditors, supervisory bodies, or regulatory authorities where it is relevant to my taking part in this research. I give permission for these individuals to have access to my records. ☐

5. I understand that relevant sections of my baby's medical notes and data collected during the study may be looked at by responsible individuals, auditors, supervisory bodies, or regulatory authorities where it is relevant to my taking part in this research. I give permission for these individuals to have access to my baby's records. ☐

6. I understand that my information will be kept confidential as required by Irish and EU law, and my personal information won't be identifiable in the database other than by a code and not my name. ☐

7. I agree that my regular doctor (GP) can be informed of my participation in the study ☐

8. I give permission for the study team to contact me in the future to collect additional information about me and my baby for future research purposes. ☐

9. I give permission for the data collected from my glucometer to be stored and analysed as part of a future observational study. ☐

10. I will receive a copy of this signed consent form and the information sheet for my records. ☐

11. I understand that my study data may be transferred within and/or outside the EU in line with reporting requirements to the drug manufacturer, MERCK. ☐

12. I confirm I have Read and Understood the information on Data Privacy and I understand my rights as outlined per GDPR and the Data Protection Bill 2018. ☐

Presenter Declaration

I have explained the nature and purpose of this research study, the procedures to be undertaken and any risks that may be involved.

Presenter (who presented/explained the document)		
Name (Print)	Signature	Date

Participant Declaration

I have read, or have read to me, this consent form. I have had the opportunity to ask questions and all my questions have been answered to my satisfaction. I freely and voluntarily agree to be part of this research study, though prejudice to my legal and ethical rights. I have received a copy of this agreement and I understand that, if there is a sponsoring company a signed copy will be sent to that sponsor. I understand that I may withdraw from the study at any time.

Participant		
Name (Print)	Signature	Date

Impartial Witness (if applicable)		
Name (Print)	Signature	Date

Declaration of Investigator's Responsibility

I have explained the nature and purpose of this research study, the procedures to be undertaken and any risks that may be involved. I have offered to answer any questions and fully answered such questions. I believe that the participant understands my explanation and has freely given informed consent.

Investigator		
Name (Print)	Signature	Date
